# Herbicide resistance—what have we learned from other disciplines?

**DOI:** 10.1007/s12154-014-0119-8

**Published:** 2014-07-30

**Authors:** Harry J. Strek

**Affiliations:** Research - Weed Control Biology, Bayer CropScience AG, Industriepark Höchst, 65926 Frankfurt, Germany

**Keywords:** Herbicide resistance, Fungicide resistance, Insecticide resistance, Best management practices

## Abstract

Herbicide resistance is a growing threat to agriculture and has parallels to resistances to fungicides and insecticides. However, there are many reasons to treat the resistance to herbicides differently. To highlight these similarities and differences, three pests, a weed, an insect, and a disease that have shown the ability to rapidly develop resistance to a variety of products and product classes were used as illustrations. The situation in herbicide resistance is approaching a point already experienced by the other pest control disciplines, and thus, it is worthwhile to revisit their experiences.

## Opinion

The recent Symposium—Biological and Chemical Approaches towards Combating Resistance in Agriculture, sponsored by RSC BMCS and AgriNet Agriscience, gave me a renewed opportunity to view presentations from colleagues working on insecticide and fungicide resistance, areas somewhat tangential but complementary to the field of herbicide resistance in which I work. I was struck by how familiar the problems were to what we face with herbicide resistant weeds, yet I also realized that some problems and issues were completely and fundamentally different. I presented a talk entitled “Herbicide Resistance and its Consequences for Agriculture and the Agrochemical Industry” that dealt in part with the impact that resistance to glyphosate in the USA by two Amaranth species, Palmer amaranth (*Amaranthus palmeri* S. Wats.) and common waterhemp (*Amaranthus tuberculatus* Moq. J.D. Sauer, formerly known as *Amaranthus rudis* L.), have had on agriculture and our industry. Due to the long germination period, rapid growth, enormous seed production capability, and strong competitiveness of these two weeds with crops [1–5], some have referred to them almost facetiously as “monster weeds” [6]. However, they are not to be taken lightly. In some areas of the USA, it is astounding to observe soybean, cotton, and corn fields during late August that are thoroughly overgrown with these weeds. Some fields are completely lost and are then mowed and plowed under, although that is not a good idea because of the return of seeds to the soil will likely flourish in next year’s crop. The spread of these Amaranths has been described befittingly as threatening to modern agriculture [7] and even as potentially resulting in near zero yield for corn and soybean [8]. The reasons for this development have been extensively reviewed [9–11], and will not be discussed herein. Why revisit now this comparison of resistance problems between the insect, disease, and weed control disciplines? It has been addressed before [12–14], but the time is right to ask some of the same questions again. What is different now is that we are currently reaching significant milestones in herbicide resistance evolution that have already been experienced before in the other disciplines, and this, coupled with the current lack of new easy fixes (i.e., herbicides with novel modes of action), is creating a sense of urgency and despair in some markets. Almost two decades ago, it was acknowledged that resistance could develop to almost any fungicide, insecticide, or acaricide and “even [a] weed” [15]. Resistance had become an everyday management problem in the management of diseases, insect pests, and nematodes, but this was not really true for weeds. Resistance by weeds to almost any herbicide was considered by some in the recent past as more of a theoretical possibility than as a real threat. This is no longer the case. The desire to learn from “their” experience is predicated partly upon the need to see if we have missed anything. While doing so, the analogy of this situation with weeds to other major resistance problems (for insects and diseases) came to mind. I wondered if a more attenuated comparison of the Amaranth resistance situation with the resistance situation of the Colorado potato beetle (*Leptinotarsa decemlineata* Say) and potato late blight (*Phytophthora infestans* (Mont.) de Bary), two pests that have had significant impacts on agriculture and food production, could not provide a practical opportunity to highlight some of the key similarities and differences at a more comprehensible level.

Certainly, we in herbicide resistance research have learned much about management practices for diseases such as potato late blight that have developed resistance rapidly to many classes of compounds. Mixtures of different modes of action may have been the most used strategy to manage resistance 20 years ago [13], but now is regularly supplemented by using complementary cultural practices, spray schedules of alternating chemistries, and “adequate” (or close to full) rates and timings as recommended on labels to lower disease pressure, and in an era of prevalent resistance, understanding and using the positive attributes of each mode of action [16, 17]. The prominent role of the industry-sponsored Resistance Action Committees (RACs), namely FRAC and IRAC, in developing coordinated, sound management practices has been instrumental in slowing resistance development in diseases and insects [18–20]. Strategies advanced by the Weed Science Society of America, HRAC, and the Australian Herbicide Resistance Initiative all agree that several concurrent steps are necessary to manage resistance, should include education about best practices and the economic benefits of proactive management [21–23], and also calls for the inclusion of disruptive practices such as harvest weed seed control [22]. We don’t necessarily know exactly how long these combinations of practices will keep herbicides working, but we do know that they need to be implemented to keep current agricultural production systems sustainable. All disciplines also agree on the need for more detailed information on pest biology, life cycles, and reproduction strategies.

One instructive excercise is to compare the biology of the Amaranths with the biology of the Colorado potato beetle. The life history of this insect has been described as being complicated and diverse and thus requiring the same kind of measures to control it, with dispersal and feeding behavior, diapause, and overwintering in soil, reproduction strategies including random mating to maximize genetic diversity, and high fecundity linked with survival of potentially devastating climate conditions or insecticide applications in a particular field [24]. It has successfully adapted itself to different habitats around the globe. The Amaranths can also be described as having a complicated and diverse life history and are also very adaptable to habitats. One major obvious difference with an insect is the sedentary nature of terrestrial plants. Insects are very mobile, plants are much less so. Palmer amaranth pollen can disperse up to 300 m in the air [25], highlighting the potential ability to disperse locally. Palmer amaranth has been found in Michigan, far outside of its normal range in the southern USA [5] likely due to long-distance seed transmission. However, pollen and occasional seed transmission are much more limited transportation vectors resulting in a restricted number of transferred resistant organisms than having practically all individuals in a Colorado potato beetle population with the ability to move easily by walking between fields for hundreds of meters or even flying up to 100 km [24]. Because of the relatively reduced mobility of weeds versus insects, it has been stated that a farmer is more in control of the weed problem that develops in his field versus being more susceptible to invasion by resistant populations of insects and diseases from neighboring fields [14]. A phylogenetic study of seven Palmer amaranth populations from North Carolina, Georgia, Tennessee, and Arkansas, resulted in some of them clustering based more upon resistance status than geography, but results were based on a low number of sampled individuals per population and could not be called conclusive [26]. Recent in-depth studies at a local landscape level of *Alopecurus myosuroides* Huds. resistant to ACCase- and ALS-inhibiting herbicides show that neighboring fields can have quite different resistance profiles, even between relatively small fields, and suggest that resistance evolved independently in the fields [27, 28]. These examples are, of course, dependent upon the particular weed species and cropping system and cannot be applied to all weeds, but it illustrates a major difference in assumptions about how resistance develops and spreads in insects and weeds. It is assumed that transport can play an important role, but relative to independent evolution, it is likely to be less important for weeds. Genetic diversity plays a role in beetle survival, as it does for the Amaranths. Both Palmer amaranth and common waterhemp are dioecious and thus obligate outcrossing species [29]. High fecundity is also a characteristic of these weeds, with reports of up to a million seeds produced per plant [30, 31]. Amaranths are C4 plants, making them efficient under high light and hot conditions, with Palmer amaranth being adapted to desert conditions and particularly suited for competition in dry situations [32]. The Colorado potato beetle has developed resistance to almost every insecticide used to control it [33]. The Amaranths are following in its footsteps, with resistance developed by each to up to six different modes of action [34]. Resistance factors (RF), generally the ratio of the ED50 or LD50 values between resistant and appropriate sensitive populations, are generally much higher in insects. For the Colorado potato beetle, a RF of 30 is not considered to be high and can reach up to 2,000 [24], whereas resistance factors for Palmer amaranth are generally below 10 [35–37]. One major difference between the *Amaranthus* weeds and the insect is in the fitness of resistant individuals. There seems to be a fitness penalty for resistant Colorado potato beetles [24] but not for Palmer amaranth [38]. Although not studied in detail, it is assumed that because of the competitiveness of resistant common waterhemp individuals growing in glyphosate-treated soybean and corn fields (personal observation), this species also does not likely possess major fitness costs associated with resistance.

We are dealing, in the USA, with two formidable weeds, Palmer amaranth and common waterhemp. Their evolutionary path has endowed them with the ability to compete effectively with crops in current agricultural production systems. They have proven to be daunting foes to agriculture, as has the Colorado potato beetle, which has been credited with helping to shape the modern pesticide industry [24]. This distinction may perhaps soon be shared with the Amaranths.
